# Association of rs10830962 polymorphism with gestational diabetes mellitus risk in a Chinese population

**DOI:** 10.1038/s41598-019-41605-3

**Published:** 2019-03-29

**Authors:** Kaipeng Xie, Ting Chen, Yue Zhang, Juan Wen, Xianwei Cui, Lianghui You, Lijun Zhu, Bo Xu, Chenbo Ji, Xirong Guo

**Affiliations:** 1 Women’s Hospital of Nanjing Medical University, The Affiliated Obstetrics and Gynecology Hospital of Nanjing Medical University, Nanjing Maternity and Child Health Care Hospital, Nanjing, China; 2 School of Information Management, Nanjing University, Nanjing, China; 3 State Key Laboratory of Reproductive Medicine, Institute of Toxicology, Nanjing Medical University, Nanjing, China

## Abstract

To date, only three polymorphisms (rs10830962, rs7754840 and rs1470579) are included in the genome-wide association study Catalog (www.ebi.ac.uk/gwas). However, the available evidence is limited in pregnant Chinese women. We aimed to explore the associations of three polymorphisms (rs10830962, rs7754840 and rs1470579) with GDM risk in a Chinese population. We conducted a case-control study (964 GDM cases and 1,021 controls) to evaluate the associations of these polymorphisms with GDM risk. A logistic regression model was used to calculate odds ratios (ORs) and their confidence intervals (CIs). After adjustment for age, prepregnancy BMI, parity, abnormal pregnancy history and family history of diabetes, the minor allele of rs10830962 (C > G) demonstrated a significant association with an increased risk of GDM (OR = 1.16, 95% CI = 1.02–1.31, *P* = 0.029 in the additive model). However, no significant association was observed between the other two polymorphisms and GDM. Subsequent functional annotation shows that rs10830962 is located in the regulatory elements of pancreatic islets, alters the binding affinity of motifs and regulates SNORA8 expression. Our findings demonstrate that rs10830962 is associated with an increased risk of GDM in the Chinese population. Further functional characterization is warranted to uncover the mechanism of the genotype-phenotype association.

## Introduction

Gestational diabetes mellitus (GDM) is defined as a glucose intolerance disorder with first onset or recognition in pregnancy that affects an estimated 14% of pregnancies globally^1^. Recently, GDM has received increasing attention due to its continuous increase in prevalence, especially in developing countries such as China and India^2^. GDM is associated with an increased risk of adverse pregnancy outcomes for pregnant women and chronic metabolic diseases for both mothers and their offspring^3–5^. It has been reported that family history of diabetes, maternal age, prepregnancy overweight, and obesity are the most common risk factors for GDM^6^. Because the occurrence of GDM is directed by multiple factors, it is critical to explore novel risk factors to identify high-risk pregnant women for early intervention.

Emerging studies have implicated genetic factors in the etiology of GDM^7,8^. Insulin secretion defects and insulin resistance are crucial in the development of GDM^9^. A study on Danish twins demonstrated that genetic components can explain over 75% of insulin secretion dysfunction and at least 53% of peripheral insulin sensitivity^10^. To date, there has been only one genome-wide association study (GWAS) for GDM^7^. In the Korean population, two single nucleotide polymorphisms (SNPs), rs10830962 and rs7754840, reached the genome-wide significance (*P* < 5 × 10^−8^), and rs1470579 demonstrated near genome-wide significance (*P* = 2.0 × 10^−7^). Accordingly, only these three GDM-associated loci are in the GWAS Catalog (www.ebi.ac.uk/gwas), which is a publicly available and manually curated resource of all published GWASs and association results^11^. Some researchers have performed candidate-gene approaches and examined the associations between the genetic polymorphisms described above and the risk of GDM. For example, Cho *et al*. confirmed that rs7754840 was associated with insulin secretory capacity and GDM risk in Koreans^12^. However, significant associations were not observed in the Egyptian and Russian populations^13,14^. Notably, the available evidence on these associations in the Chinese population is quite limited^15,16^. Based on a case-control study that included 350 GDM patients and 480 control subjects, Li *et al*. concluded that rs10830962 was not associated with the development of GDM in a Chinese population^17^. A relationship between rs7754840 and GDM was also not observed in a Chinese population^18^. In particular, the effect of rs1470579 on the susceptibility to GDM has not been explored in Chinese pregnant women.

Given the different genetic backgrounds and the inadequate evidence about the effect of these three polymorphisms on GDM risk, we conducted a case-control study to determine whether these polymorphisms contribute to GDM risk in a Chinese population.

## Results

### Subject characteristics

The demographic characteristics of the 964 GDM patients and 1,021 controls are summarized in Table 1. The ages and prepregnancy body mass index (BMI) values were comparable between cases and controls (*P*_age_ = 0.118, *P*_Prepregnancy BMI_ = 0.408). The GDM cases had higher rates of multiparity (14.21%), abnormal pregnancy history (12.14%) and family history of diabetes (17.95%) than the controls (all *P* < 0.05).

**Table 1:** Characteristics of subjects.

Variables	Cases (N = 964)	Controls (N = 1021)	*P*
	N (%)	N (%)	
Age (years)	30.57 ± 3.69*	30.30 ± 3.59*	0.094
<35	827 (85.79)	900 (88.15)	0.118
≥35	137 (14.21)	121 (11.85)	
Prepregnancy BMI (kg/m^2^)	22.08 ± 2.94*	22.03 ± 2.83*	0.685
Underweight, 18.5	83 (8.61)	100 (9.79)	0.408
Normal weight, 18.5–23.9	657 (68.15)	691 (67.68)	
Overweight, 24.0–27.9	184 19.09)	200 (19.59)	
Obese, ≥28	40 (4.15)	30 (2.94)	
Parity			
Nulliparous	827 (85.79)	953 (93.34)	<**0.001**
Multiparous	137 (14.21)	68 (6.66)	
Abnormal pregnancy history			
No	847 (87.86)	981 (96.08)	<**0.001**
Yes	117 (12.14)	40 (3.92)	
Family history of diabetes			
No	791 (82.05)	876 (85.80)	**0.023**
Yes	173 (17.95)	145 (14.20)	

*Mean ± SD.

### Associations of three SNPs with GDM risk

The genotype distributions of the three SNPs between cases and controls are shown in Supplementary Table S1. The genotype frequencies of the three SNPs were all in Hardy-Weinberg equilibrium among the controls (all *P* > 0.05). Table 2 summarizes these variant associations under codominant, dominant, recessive and additive models. After adjustment for age, prepregnancy BMI, parity, abnormal pregnancy history and family history of diabetes, the minor allele of rs10830962 (C > G) showed a significant association with an increased risk of GDM [additive model: Odds ratio (OR) 95% confidence interval (CI) = 1.16 (1.02–1.31), *P* = 0.029, Table 2]. The results were still robust under the recessive model [OR (95% CI) = 1.30 (1.04–1.63), *P* = 0.023] and a codominant model [GG vs. CC, OR (95% CI) = 1.36 (1.04–1.76), *P* = 0.022]. However, no significant association was observed between the other two SNPs and GDM risk in any model. To better understand the effect of rs10830962 on the risk of GDM, we performed stratified analyses based on age, prepregnancy BMI, parity, abnormal pregnancy history and family history of diabetes; however, no significant differences were observed among these subgroups (homogeneity test *P* > 0.05 for all comparisons, Table 3).

**Table 2:** Association of three SNPs with GDM risk.

Genotype	Cases (N = 964)^a^	Controls (N = 1021)^a^	Adjusted OR	*P* ^c^
	N	N	(95% CI)^c^	
rs10830962 (C > G)				
CC	278	316	1.00	
CG	468	504	1.07 (0.87–1.32)	0.518
GG	206	182	**1.36 (1.04–1.76)**	**0.022**
Dominant model				
CC	278	316	1.00	
CG/GG	674	686	1.15 (0.94–1.40)	0.175
Recessive model				
CC/CG	746	820	1.00	
GG	206	182	**1.30 (1.04–1.63)**	**0.023**
Additive model	—	—	**1.16 (1.02–1.31)**	**0.029**
rs1470579 (A > C)				
AA	507	546	1.00	
AC	371	401	0.97 (0.80–1.17)	0.742
CC	71	52	1.42 (0.97–2.09)	0.073
Dominant model				
AA	507	546	1.00	
AC/CC	442	453	1.02 (0.85–1.22)	0.824
Recessive model				
AA/AC	878	947	1.00	
CC	71	52	1.44 (0.99–2.10)	0.057
Additive model	—	—	1.07 (0.93–1.24)	0.349
rs7754840 (G > C)				
GG	310	353		
GC	449	461	1.09 (0.89–1.33)	0.420
CC	185	182	1.15 (0.89–1.50)	0.284
Dominant model				
GG	310	353	1.00	
GC/CC	634	643	1.11 (0.91–1.34)	0.302
Recessive model				
GG/GC	759	814	1.00	
CC	185	182	1.10 (0.87–1.38)	0.429
Additive model	—	—	1.08 (0.95–1.22)	0.262

^a^Major homozygote/heterozygote/minor homozygote.

^b^MAF, minor allele frequency among controls.

^c^Adjusted by age, prepregnancy BMI, parity, abnormal pregnancy history and family history of diabetes.

**Table 3:** Stratified analysis of rs10830962 genotypes associated with GDM risk.

Variables	rs10830962 (CC/CG/GG)		Adjusted OR	*P* for
	Cases^a^	Controls^a^	(95% CI)^b^	heterogeneity
Age (year)				
<35	240/401/175	277/444/163	**1.15 (1.00–1.32)**	0.643
≥35	38/67/31	39/60/19	1.26 (0.88–1.81)	
Prepregnancy BMI (kg/m^2^)				
Underweight, 18.5	19/39/24	31/46/22	1.37 (0.90–2.07)	0.855
Normal weight, 18.5–23.9	189/326/135	207/353/117	1.16 (0.98–1.36)	
Overweight, 24.0–27.9	57/85/40	66/94/36	1.11 (0.83–1.49)	
Obese, ≥28	13/18/7	12/11/7	1.05 (0.53–2.10)	
Parity				
Nulliparous	234/400/181	295/469/170	**1.17 (1.02–1.34)**	0.348
Multiparous	44/68/25	21/35/12	0.93 (0.59–1.48)	
Abnormal pregnancy history				
No	237/406/192	303/486/174	**1.18 (1.03–1.35)**	0.255
Yes	41/62/14	13/18/8	0.83 (0.46–1.50)	
Family history of diabetes				
No	225/388/167	275/432/152	**1.19 (1.03–1.37)**	0.183
Yes	53/80/39	41/72/30	0.94 (0.69–1.30)	

^a^Major homozygote/heterozygote/minor homozygote.

^b^Adjusted by age, prepregnancy BMI, parity, abnormal pregnancy history and family history of diabetes except for the stratified factor.

### Functional annotation of rs10830962

Based on the Encyclopedia of DNA Elements (ENCODE) and Roadmap databases, we found that rs10830962 is located in functional regulatory elements of human pancreatic islets, such as those with high DNaseI hypersensitivity (DNaseI HS) density signals and Formaldehyde-Assisted Isolation of Regulatory Elements (FAIRE) density signals and several histone modification markers including H3K27ac, H3K36me3, and H3K4me1 (Supplementary Fig. S1). Using the HaploReg tool, we found that the rs10830962 G allele could increase the binding affinity of DMRT5, FOXL1, HMBOX 1 and PU.1 while decreasing the binding affinity of HNF1 and MEF2 (Supplementary Table S2). We further consulted PhenoScanner and found that rs10830962 could significantly regulate the expression levels of several genes including SNORA8, SCARNA9, FAT3, TAF1D, SNORA25, SNORA18, SNORA32, C11orf54, SLC36A4 and MED17 in multiple tissues (Supplementary Table S3). These tissues include visceral omental adipose tissue, the anterior cingulate cortex, the cerebellum, the hippocampus, the nucleus accumbens, mammary tissue, transformed fibroblasts, the sigmoid colon, the esophageal mucosa, the tibial nerve, the pancreas, and sun-exposed lower leg skin tissue. Interestingly, rs10830962 and its correlated variants within a linkage disequilibrium (LD) block could significantly regulate the expression levels of SNORA8 in pancreas tissue, as described in Table 4.

**Table 4:** rs10830962 and its high-LD (r^2^ > 0.80) SNPs and SNORA8 gene expression in human pancreas tissue.

SNP	Proxy rsID	Proxy Alleles	r^2^	Source	Tissue	Gene	N	Effect Allele	Association Alleles	Beta	SE	*P*
rs10830962	rs10830962	C/G	1.00	GTEx	Pancreas	SNORA8	149	C	C/G	0.252	0.093	**0.008**
rs10830962	rs4331050	G/T	1.00	GTEx	Pancreas	SNORA8	149	G	G/T	0.248	0.092	**0.008**
rs10830962	rs7941837	A/T	0.96	GTEx	Pancreas	SNORA8	149	A	A/T	0.240	0.089	**0.008**
rs10830962	rs7945617	T/C	0.96	GTEx	Pancreas	SNORA8	149	T	T/C	0.242	0.089	**0.008**
rs10830962	rs10466351	C/T	0.86	GTEx	Pancreas	SNORA8	149	C	C/T	0.249	0.084	**0.004**

## Discussion

We examined the associations of 3 SNPs with the risk of GDM in a Chinese population. Our findings suggest that rs10830962 (C > G) confers an increased risk of developing GDM. Further functional annotation indicated that rs10830962 (C > G) alters the binding of multiple motifs and alters the expression levels of SNORA8 in pancreas tissue. These findings indicate that the polymorphism may participate in the pathogenesis of GDM.

Our findings confirmed that the minor allele of rs10830962 increased the GDM risk in a Chinese population, which is consistent with the result in the Korean population^7^. In a previous study, the minor allele of rs10830962 was associated with decreased fasting insulin concentrations in women with GDM^7^. It has been reported that this polymorphism also determines glucose-stimulated insulin secretion and plasma glucose concentrations and thus increases the type 2 diabetes mellitus (T2DM) risk in European populations^19^. As decreased beta-cell insulin secretory function plays a central role in both GDM and type 2 diabetes, it is conceivable that rs10830962 might affect beta-cell function in the pathogenesis of GDM. Rs10830962 is located 4.4 kb upstream of MTNR1B. Bioinformatics analyses revealed that rs10830962 is located in the functional elements of pancreatic islets and alters motif binding. Among the altered motifs, HNF1, a predominant trans-acting factor of hepatic or pancreaticbeta-cells, targets many genes involved in carbohydrate metabolism^20^. The binding of the HNF1 motif could directly activate beta-cell genes and directly influence glucose-stimulated insulin secretion in pancreatic beta-cells^21,22^. Considering these findings, we speculate that the C to G base change of rs10830962 may disturb HNF1 binding, regulate beta-cell gene expression, and thus have deleterious effects on beta-cell function. Unfortunately, we did not observe a relationship between rs10830962 and the expression of the nearest gene, MTNR1B. We observed that rs10830962 and its correlated variants were significantly associated with SNORA8 expression. These findings suggested that these SNPs were involved in the regulation of SNORA8 expression and thus contributed to the development of GDM. However, there have been no studies about the role of SNORA8 in beta-cell function or insulin secretion. Therefore, it is worth investigating the functional role of rs10830962 and SNORA8 in the pathogenesis of GDM with functional assays.

Our results differ from those of Li *et al*., who suggested that rs10830962 is not associated with any risk of developing GDM in pregnant Chinese women^17^. The lack of an association of rs10830962 with GDM in the earlier study may be attributed to the limited adjustment factors, including age and prepregnancy BMI, and a small sample size. In addition, there was no significant association between the other two SNPs (rs7754840 and rs1470579) and GDM risk in our study. Consistent with our findings, another study in pregnant Chinese women found no significant association between rs7754840 and GDM risk^18^, whereas studies in Korean, Caucasian and South Indian populations showed significant associations between rs7754840 and GDM risk^12,16,23^. This discrepancy could be due to differences in ethnicities, sample sizes and diagnostic criteria for GDM. Although have been no studies about the effect of rs1470579 on GDM risk, in one recent meta-analysis of 36 studies by Ping *et al*., researchers demonstrated that rs1470579 was associated with T2DM risk in Asians^24^. However, it was shown that rs1470579 did not predict the development of postpartum diabetes in women with GDM^25^.

In our study, we attempted to reduce the potential confounding bias. Cases and controls were matched for age and prepregnancy BMI. We adjusted for other factors, including parity, abnormal pregnancy history and family history of diabetes. Nevertheless, we did not consider other GDM-associated factors, such as food intake and physical activity^26,27^. Further studies are needed to assess the relationship after adjustment for these factors.

In summary, our study provides evidence that rs10830962 is significantly associated with GDM risk in pregnant Chinese women, highlighting the importance of this potentially functional variant in GDM development. Functional investigations are needed to discover the underlying mechanisms.

## Materials and Methods

### Study population

This study was carried out according to the guidelines in the Declaration of Helsinki and all procedures were approved by the institutional review board of Nanjing Maternity and Child Health Care Hospital. Based on a study population of over 80,000 women who attended pregnancy complications screening between March 2012 and February 2015 at the Nanjing Maternity and Child Health Care Hospital, a total of 964 GDM cases and 1,021 controls were randomly selected as previously described^28^. All participants were offered a glucose challenge test (GCT) at 24–28 weeks of gestation and gave written informed consent at recruitment. We excluded women who had diabetes before pregnancy from our study. GDM was defined as fasting glucose ≥5.5 mmol/L or a 2-hour plasma glucose ≥8.0 mmol/L following a 75-g oral glucose tolerance test (OGTT)^29^. The controls were pregnant women without diabetes or previous metabolic diseases and were frequency-matched to GDM cases on age and prepregnancy BMI. We collected participants’ demographics information, including maternal age, prepregnancy height and weight, parity, abnormal pregnancy history and family history of diabetes, from their medical records and subsequent interviews.

### SNP selection and genotyping

Three polymorphisms (rs10830962, rs7754840 and rs1470579) reported in GDM GWASs were included. Based on the 1000 Genomes Project Phase I, all the polymorphisms had a minor allele frequency (MAF) greater than 0.05 and did not have strong LD (R^2^ > 0.80) in the Chinese Han population. As a result, all three SNPs were genotyped in our study.

We used the proteinase K digestion and phenol/chloroform extraction method to extract genomic DNA and then diluted the DNA to working concentrations (20 ng/μL) for genotyping. SNPs were genotyped with the Sequenom MassARRAY platform (Sequenom, San Diego, CA).

### In silico analysis

To further elucidate the function of significant polymorphisms in the pathogenesis of GDM, we used the ENCODE (http://genome.ucsc.edu/encode/) database and the Roadmap Epigenomics Project (http://genomebrowser.wustl.edu/) database to explore whether the SNPs were located in functional elements. Subsequently, HaploReg V4 (http://compbio.mit.edu/HaploReg) was used to examine the significant SNPs and the loci in high LD (R^2^ > 0.8 in Asian from the 1000 Genomes Project) for functional elements available in ChromHMM software (core 15-state model)^30^. Moreover, we queried the associated SNPs and their high-LD SNPs (r^2^ > 0.8 from the 1000 Genomes Project) against the PhenoScanner database to investigate the genotype-phenotype associations and extracted all significant associations for expression quantitative trait loci (eQTL) analysis^31^.

### Statistical analysis

Differences in the distribution of demographic characteristics between GDM cases and controls were calculated by the χ^2^ test (for categorical variables) or Student’s t-test (for continuous variables). Genotype frequencies in controls were tested for Hardy-Weinberg equilibrium (HWE) by the goodness-of-fit χ^2^ test. ORs and their 95% CIs were calculated using logistic regression analysis to assess the associations between genotypes and GDM risk after adjusting for age, prepregnancy BMI, parity, abnormal pregnancy history and family history of diabetes. The χ^2^-based Q test was used to evaluate the heterogeneity of associations between subgroups. All statistical analyses were performed using PLINK software (V1.07) and R software (version 3.2.5). A two-sided *P* < 0.05 was considered statistically significant.

## Supplementary information

Supplementary Tables_Figures

## Data Availability

The genotype dataset in the current study has been deposited at figshare (10.6084/m9.figshare.7743326). Due to user privacy, the dataset of baseline information in the current study is available from chenboji@njmu.edu.cn on reasonable request. Data that support the findings of this study are available from PhenoScanner database (http://www.phenoscanner.medschl.cam.ac.uk).
