# Supplementary material for: Association of rs10830962 polymorphism with gestational diabetes mellitus risk in a Chinese population
Source: Sci Rep. 2019 Mar 29;9:5357. doi: 10.1038/s41598-019-41605-3 (PMC6440982; doi:10.1038/s41598-019-41605-3)
Supplement: Supplementary file 1 — Supplementary Tables_Figures [file 41598_2019_41605_MOESM1_ESM.pdf]

Association of rs10830962 polymorphism with gestational diabetes mellitus risk in a Chinese population

Kaipeng Xie[1†], Ting Chen[1†], Yue Zhang[2], Juan Wen[1], Xianwei Cui[1], Lianghui You[1], Lijun Zhu[1], Bo Xu[3], Chenbo Ji[1*], Xirong Guo[1*]

[1] The Affiliated Obstetrics and Gynecology Hospital of Nanjing Medical University, Nanjing Maternity and Child Health Care Hospital, Nanjing, China

[2] School of Information Management, Nanjing University, Nanjing, China

[3] State Key Laboratory of Reproductive Medicine, Institute of Toxicology, Nanjing Medical University, Nanjing, China

[†] These authors contributed equally to this work.

[*]**Corresponding author**: Xirong Guo, Nanjing Maternity and Child Health Care Institute, Nanjing Maternity and Child Health Care Hospital Affiliated to Nanjing Medical University, Nanjing, 210004, China, Tel (Fax): 86-25-8446-0507, E-mail: xrguo@njmu.edu.cn, or Chenbo Ji, Nanjing Maternity and Child Health Care Institute, Nanjing Maternity and Child Health Care Hospital Affiliated to Nanjing Medical University, Nanjing, 210004, China, Tel (Fax): 86-25-8446-0507, E-mail: chenboji@njmu.edu.cn.

Supplementary Table S1. Genotyping results of three SNPs in GDM cases and controls

SNP	Allele[a]	MAF[b]	MAF[c]	HWE[d]	Call rate	Cases[e] N = 964	Control[e] N=1021
rs1470579	A/C	0.289	0.253	0.054	98.14%	507/371/71	546/401/52
rs7754840	G/C	0.428	0.414	0.151	97.73%	310/449/185	353/461/182
rs10830962	C/G	0.438	0.433	0.479	98.44%	278/468/206	316/504/182

[a]Major/minor allele

[b]MAF, minor allele frequency in Chinese Han population based on the 1000 Genomes Project.

[c]MAF, minor allele frequency in controls.

[d]HWE, Hardy-Weinberg equilibrium in controls

[e] Major homozygote/heterozygote/minor homozygote

Supplementary Table S2. Summary of genomic annotation by HaploReg v4 for SNPs with rs10830962 in high LD (r^2 > 0.80)

Chr	LD (r^2)	Variants	ASN freq	Motifs changed	RefSeq genes
11	0.99	rs4331050	0.45	5 altered motifs [a]	6.8kb 5' of MTNR1B
11	1.00	rs10466351	0.45	HNF1, Hsf, ZEB1	4.8kb 5' of MTNR1B
**11**	**1.00**	**rs10830962**	**0.45**	**6 altered motifs [b]**	4.4kb 5' of MTNR1B
11	0.99	rs7941837	0.45	AP-1, THAP1	3.1kb 5' of MTNR1B
11	0.87	rs7945617	0.48	NF-kappaB, Nkx2	2.5kb 5' of MTNR1B
11	0.88	rs10830963	0.44	Eomes,TAL1,YY1	MTNR1B

[b] The altered motifs included the increased binding affinity of FAC1, Foxa_known2, Foxp1, HDAC2_disc6, Zfp105 and the decreased binding affinity of Foxa_known4.

[b] The altered motifs included the increased binding affinity of DMRT5, Foxl1_1, HNF1_6, Hmbox1 and PU.1_disc1, and the decreased binding affinity of HNF1_1 and Mef2_know5.

Supplementary Table S3. Detailed functional annotation of rs10830962 and its high LD (r2 > 0.80) using PhenoScanner

SNP	Proxy rsID	Proxy Alleles	r^2	Source	Tissue	Gene	N	Effect Allele	Beta	SE	*P*
rs10830962	rs10830962	C/G	1.00	GTEx	Adipose visceral omentum	SCARNA9	185	C	-0.24	0.08	0.0033
rs10830962	rs4331050	G/T	1.00	GTEx	Adipose visceral omentum	SCARNA9	185	G	-0.23	0.08	0.0041
rs10830962	rs7941837	A/T	0.96	GTEx	Adipose visceral omentum	SCARNA9	185	A	-0.23	0.08	0.0030
rs10830962	rs7945617	T/C	0.96	GTEx	Adipose visceral omentum	SCARNA9	185	T	-0.23	0.08	0.0037
rs10830962	rs10830961	A/G	0.96	GTEx	Adipose visceral omentum	SCARNA9	185	A	-0.23	0.08	0.0047
rs10830962	rs7941837	A/T	0.96	GTEx	Adrenal gland	CCDC67	126	A	0.25	0.09	0.0058
rs10830962	rs7945617	T/C	0.96	GTEx	Adrenal gland	CCDC67	126	T	0.25	0.09	0.0058
rs10830962	rs10466351	C/T	0.86	GTEx	Adrenal gland	CCDC67	126	C	0.24	0.08	0.0059
rs10830962	rs7933855	G/A	0.87	GTEx	Adrenal gland	VSTM5	126	G	-0.30	0.11	0.0100
rs10830962	rs10830961	A/G	0.96	GTEx	Artery aorta	KIAA1731	197	A	0.17	0.06	0.0055
rs10830962	rs2166706	T/C	0.84	GTEx	Artery aorta	KIAA1731	197	T	0.18	0.06	0.0033
rs10830962	rs724030	A/G	0.84	GTEx	Artery aorta	KIAA1731	197	A	0.17	0.06	0.0041
rs10830962	rs2166704	T/A	0.84	GTEx	Artery aorta	KIAA1731	197	T	0.17	0.06	0.0043
rs10830962	rs10765575	A/C	0.84	GTEx	Artery aorta	KIAA1731	197	A	0.17	0.06	0.0059
rs10830962	rs2895443	G/A	0.84	GTEx	Artery aorta	KIAA1731	197	G	0.17	0.06	0.0042
rs10830962	rs10830962	C/G	1.00	GTEx	Brain anterior cingulate cortex ba24	SNORA8	72	C	-0.23	0.08	0.0064
rs10830962	rs4331050	G/T	1.00	GTEx	Brain anterior cingulate cortex ba24	SNORA8	72	G	-0.22	0.08	0.0094
rs10830962	rs7941837	A/T	0.96	GTEx	Brain anterior cingulate cortex ba24	SNORA8	72	A	-0.23	0.08	0.0058
rs10830962	rs7945617	T/C	0.96	GTEx	Brain anterior cingulate cortex ba24	SNORA8	72	T	-0.23	0.08	0.0063
rs10830962	rs10830961	A/G	0.96	GTEx	Brain anterior cingulate cortex ba24	SNORA8	72	A	-0.21	0.08	0.0097
rs10830962	rs7948640	C/T	0.89	GTEx	Brain anterior cingulate cortex ba24	SNORA8	72	C	-0.24	0.08	0.0045
rs10830962	rs7948632	C/T	0.89	GTEx	Brain anterior cingulate cortex ba24	SNORA8	72	C	-0.24	0.08	0.0045
rs10830962	rs11020123	C/T	0.87	GTEx	Brain anterior cingulate cortex ba24	SNORA8	72	C	-0.27	0.08	0.0018

rs10830962	rs7933855	G/A	0.87	GTEx	Brain anterior cingulate cortex ba24	SNORA8	72	G	-0.25	0.08	0.0028
rs10830962	rs1985254	T/C	0.86	GTEx	Brain anterior cingulate cortex ba24	SNORA8	72	T	-0.26	0.08	0.0029
rs10830962	rs2121649	C/T	0.86	GTEx	Brain anterior cingulate cortex ba24	SNORA8	72	C	-0.25	0.08	0.0031
rs10830962	rs10466351	C/T	0.86	GTEx	Brain anterior cingulate cortex ba24	SNORA8	72	C	-0.23	0.08	0.0037
rs10830962	rs11020116	A/C	0.86	GTEx	Brain anterior cingulate cortex ba24	SNORA8	72	A	-0.25	0.08	0.0028
rs10830962	rs2166706	T/C	0.84	GTEx	Brain anterior cingulate cortex ba24	SNORA8	72	T	-0.24	0.08	0.0053
rs10830962	rs724030	A/G	0.84	GTEx	Brain anterior cingulate cortex ba24	SNORA8	72	A	-0.24	0.08	0.0053
rs10830962	rs2166704	T/A	0.84	GTEx	Brain anterior cingulate cortex ba24	SNORA8	72	T	-0.25	0.08	0.0028
rs10830962	rs10765575	A/C	0.84	GTEx	Brain anterior cingulate cortex ba24	SNORA8	72	A	-0.25	0.08	0.0030
rs10830962	rs2895443	G/A	0.84	GTEx	Brain anterior cingulate cortex ba24	SNORA8	72	G	-0.25	0.08	0.0030
rs10830962	rs1985254	T/C	0.86	GTEx	Brain caudate basal ganglia	CCDC67	100	T	-0.24	0.09	0.0076
rs10830962	rs2166706	T/C	0.84	GTEx	Brain caudate basal ganglia	CCDC67	100	T	-0.24	0.09	0.0098
rs10830962	rs724030	A/G	0.84	GTEx	Brain caudate basal ganglia	CCDC67	100	A	-0.24	0.09	0.0098
rs10830962	rs10830962	C/G	1.00	GTEx	Brain cerebellum	FAT3	103	C	0.17	0.05	0.0008
rs10830962	rs4331050	G/T	1.00	GTEx	Brain cerebellum	FAT3	103	G	0.17	0.05	0.0009
rs10830962	rs7941837	A/T	0.96	GTEx	Brain cerebellum	FAT3	103	A	0.17	0.05	0.0006
rs10830962	rs7945617	T/C	0.96	GTEx	Brain cerebellum	FAT3	103	T	0.18	0.05	0.0004
rs10830962	rs10830961	A/G	0.96	GTEx	Brain cerebellum	FAT3	103	A	0.17	0.05	0.0008
rs10830962	rs7948640	C/T	0.89	GTEx	Brain cerebellum	FAT3	103	C	0.17	0.05	0.0028
rs10830962	rs7948632	C/T	0.89	GTEx	Brain cerebellum	FAT3	103	C	0.17	0.05	0.0028
rs10830962	rs11020123	C/T	0.87	GTEx	Brain cerebellum	FAT3	103	C	0.18	0.05	0.0011
rs10830962	rs7933855	G/A	0.87	GTEx	Brain cerebellum	FAT3	103	G	0.18	0.05	0.0011
rs10830962	rs1985254	T/C	0.86	GTEx	Brain cerebellum	FAT3	103	T	0.18	0.05	0.0010
rs10830962	rs2121649	C/T	0.86	GTEx	Brain cerebellum	FAT3	103	C	0.17	0.05	0.0012
rs10830962	rs10466351	C/T	0.86	GTEx	Brain cerebellum	FAT3	103	C	0.15	0.05	0.0026
rs10830962	rs11020116	A/C	0.86	GTEx	Brain cerebellum	FAT3	103	A	0.18	0.05	0.0011
rs10830962	rs2166706	T/C	0.84	GTEx	Brain cerebellum	FAT3	103	T	0.16	0.05	0.0039

rs10830962	rs724030	A/G	0.84	GTEx	Brain cerebellum	FAT3	103	A	0.16	0.05	0.0039
rs10830962	rs2166704	T/A	0.84	GTEx	Brain cerebellum	FAT3	103	T	0.15	0.05	0.0044
rs10830962	rs10765575	A/C	0.84	GTEx	Brain cerebellum	FAT3	103	A	0.15	0.05	0.0043
rs10830962	rs2895443	G/A	0.84	GTEx	Brain cerebellum	FAT3	103	G	0.16	0.05	0.0034
rs10830962	rs10830962	C/G	1.00	GTEx	Brain hippocampus	SNORA8	81	C	-0.22	0.08	0.0064
rs10830962	rs4331050	G/T	1.00	GTEx	Brain hippocampus	SNORA8	81	G	-0.21	0.08	0.0097
rs10830962	rs7941837	A/T	0.96	GTEx	Brain hippocampus	SNORA8	81	A	-0.22	0.08	0.0067
rs10830962	rs7945617	T/C	0.96	GTEx	Brain hippocampus	SNORA8	81	T	-0.22	0.08	0.0052
rs10830962	rs7948640	C/T	0.89	GTEx	Brain hippocampus	SNORA8	81	C	-0.21	0.08	0.0079
rs10830962	rs7948632	C/T	0.89	GTEx	Brain hippocampus	SNORA8	81	C	-0.21	0.08	0.0079
rs10830962	rs2166706	T/C	0.84	GTEx	Brain hippocampus	SNORA8	81	T	-0.23	0.08	0.0059
rs10830962	rs724030	A/G	0.84	GTEx	Brain hippocampus	SNORA8	81	A	-0.23	0.08	0.0059
rs10830962	rs2166704	T/A	0.84	GTEx	Brain hippocampus	SNORA8	81	T	-0.21	0.08	0.0097
rs10830962	rs10765575	A/C	0.84	GTEx	Brain hippocampus	SNORA8	81	A	-0.22	0.08	0.0086
rs10830962	rs10830961	A/G	0.96	GTEx	Brain nucleus accumbens basal ganglia	MED17	93	A	-0.25	0.10	0.0100
rs10830962	rs11020123	C/T	0.87	GTEx	Brain nucleus accumbens basal ganglia	SNORA18	93	C	-0.32	0.12	0.0080
rs10830962	rs2895443	G/A	0.84	GTEx	Brain nucleus accumbens basal ganglia	SNORA18	93	G	-0.35	0.12	0.0060
rs10830962	rs10830962	C/G	1.00	GTEx	Brain nucleus accumbens basal ganglia	TAF1D	93	C	-0.32	0.07	0.0000
rs10830962	rs4331050	G/T	1.00	GTEx	Brain nucleus accumbens basal ganglia	TAF1D	93	G	-0.32	0.07	0.0000
rs10830962	rs7941837	A/T	0.96	GTEx	Brain nucleus accumbens basal ganglia	TAF1D	93	A	-0.30	0.07	0.0001
rs10830962	rs7945617	T/C	0.96	GTEx	Brain nucleus accumbens basal ganglia	TAF1D	93	T	-0.30	0.07	0.0001
rs10830962	rs10830961	A/G	0.96	GTEx	Brain nucleus accumbens basal ganglia	TAF1D	93	A	-0.30	0.07	0.0001
rs10830962	rs7948640	C/T	0.89	GTEx	Brain nucleus accumbens basal ganglia	TAF1D	93	C	-0.30	0.08	0.0003
rs10830962	rs7948632	C/T	0.89	GTEx	Brain nucleus accumbens basal ganglia	TAF1D	93	C	-0.30	0.08	0.0003
rs10830962	rs11020123	C/T	0.87	GTEx	Brain nucleus accumbens basal ganglia	TAF1D	93	C	-0.30	0.08	0.0002
rs10830962	rs7933855	G/A	0.87	GTEx	Brain nucleus accumbens basal ganglia	TAF1D	93	G	-0.30	0.08	0.0002
rs10830962	rs1985254	T/C	0.86	GTEx	Brain nucleus accumbens basal ganglia	TAF1D	93	T	-0.29	0.08	0.0004

rs10830962	rs2121649	C/T	0.86	GTEx	Brain nucleus accumbens basal ganglia	TAF1D	93	C	-0.29	0.08	0.0003
rs10830962	rs10466351	C/T	0.86	GTEx	Brain nucleus accumbens basal ganglia	TAF1D	93	C	-0.30	0.07	0.0001
rs10830962	rs11020116	A/C	0.86	GTEx	Brain nucleus accumbens basal ganglia	TAF1D	93	A	-0.30	0.08	0.0002
rs10830962	rs2166706	T/C	0.84	GTEx	Brain nucleus accumbens basal ganglia	TAF1D	93	T	-0.27	0.08	0.0010
rs10830962	rs724030	A/G	0.84	GTEx	Brain nucleus accumbens basal ganglia	TAF1D	93	A	-0.27	0.08	0.0010
rs10830962	rs2166704	T/A	0.84	GTEx	Brain nucleus accumbens basal ganglia	TAF1D	93	T	-0.28	0.08	0.0006
rs10830962	rs10765575	A/C	0.84	GTEx	Brain nucleus accumbens basal ganglia	TAF1D	93	A	-0.28	0.08	0.0007
rs10830962	rs2895443	G/A	0.84	GTEx	Brain nucleus accumbens basal ganglia	TAF1D	93	G	-0.29	0.08	0.0004
rs10830962	rs10830962	C/G	1.00	GTEx	Breast mammary tissue	SNORA25	183	C	0.16	0.06	0.0094
rs10830962	rs10830961	A/G	0.96	GTEx	Breast mammary tissue	SNORA25	183	A	0.17	0.06	0.0062
rs10830962	rs7948640	C/T	0.89	GTEx	Breast mammary tissue	SNORA25	183	C	0.17	0.06	0.0070
rs10830962	rs7948632	C/T	0.89	GTEx	Breast mammary tissue	SNORA25	183	C	0.17	0.06	0.0070
rs10830962	rs724030	A/G	0.84	GTEx	Breast mammary tissue	SNORA25	183	A	0.16	0.06	0.0097
rs10830962	rs2166704	T/A	0.84	GTEx	Breast mammary tissue	SNORA25	183	T	0.16	0.06	0.0097
rs10830962	rs2895443	G/A	0.84	GTEx	Breast mammary tissue	SNORA25	183	G	0.16	0.06	0.0082
rs10830962	rs7948640	C/T	0.89	GTEx	Breast mammary tissue	SNORA40	183	C	0.22	0.08	0.0079
rs10830962	rs7948632	C/T	0.89	GTEx	Breast mammary tissue	SNORA40	183	C	0.22	0.08	0.0079
rs10830962	rs10830962	C/G	1.00	GTEx	Cells transformed fibroblasts	SNORA18	272	C	-0.14	0.04	0.0005
rs10830962	rs4331050	G/T	1.00	GTEx	Cells transformed fibroblasts	SNORA18	272	G	-0.14	0.04	0.0007
rs10830962	rs7941837	A/T	0.96	GTEx	Cells transformed fibroblasts	SNORA18	272	A	-0.12	0.04	0.0020
rs10830962	rs7945617	T/C	0.96	GTEx	Cells transformed fibroblasts	SNORA18	272	T	-0.12	0.04	0.0021
rs10830962	rs10830961	A/G	0.96	GTEx	Cells transformed fibroblasts	SNORA18	272	A	-0.11	0.04	0.0071
rs10830962	rs7948640	C/T	0.89	GTEx	Cells transformed fibroblasts	SNORA18	272	C	-0.14	0.04	0.0008
rs10830962	rs7948632	C/T	0.89	GTEx	Cells transformed fibroblasts	SNORA18	272	C	-0.14	0.04	0.0007
rs10830962	rs11020123	C/T	0.87	GTEx	Cells transformed fibroblasts	SNORA18	272	C	-0.15	0.04	0.0002
rs10830962	rs7933855	G/A	0.87	GTEx	Cells transformed fibroblasts	SNORA18	272	G	-0.13	0.04	0.0013
rs10830962	rs1985254	T/C	0.86	GTEx	Cells transformed fibroblasts	SNORA18	272	T	-0.13	0.04	0.0016

rs10830962	rs2121649	C/T	0.86	GTEx	Cells transformed fibroblasts	SNORA18	272	C	-0.15	0.04	0.0003
rs10830962	rs10466351	C/T	0.86	GTEx	Cells transformed fibroblasts	SNORA18	272	C	-0.12	0.04	0.0016
rs10830962	rs11020116	A/C	0.86	GTEx	Cells transformed fibroblasts	SNORA18	272	A	-0.15	0.04	0.0003
rs10830962	rs2166706	T/C	0.84	GTEx	Cells transformed fibroblasts	SNORA18	272	T	-0.11	0.04	0.0056
rs10830962	rs724030	A/G	0.84	GTEx	Cells transformed fibroblasts	SNORA18	272	A	-0.12	0.04	0.0036
rs10830962	rs2166704	T/A	0.84	GTEx	Cells transformed fibroblasts	SNORA18	272	T	-0.12	0.04	0.0022
rs10830962	rs10765575	A/C	0.84	GTEx	Cells transformed fibroblasts	SNORA18	272	A	-0.11	0.04	0.0060
rs10830962	rs2895443	G/A	0.84	GTEx	Cells transformed fibroblasts	SNORA18	272	G	-0.13	0.04	0.0014
rs10830962	rs10830962	C/G	1.00	GTEx	Cells transformed fibroblasts	SNORA32	272	C	-0.21	0.06	0.0013
rs10830962	rs4331050	G/T	1.00	GTEx	Cells transformed fibroblasts	SNORA32	272	G	-0.20	0.06	0.0014
rs10830962	rs7941837	A/T	0.96	GTEx	Cells transformed fibroblasts	SNORA32	272	A	-0.19	0.06	0.0035
rs10830962	rs7945617	T/C	0.96	GTEx	Cells transformed fibroblasts	SNORA32	272	T	-0.19	0.06	0.0030
rs10830962	rs10830961	A/G	0.96	GTEx	Cells transformed fibroblasts	SNORA32	272	A	-0.20	0.06	0.0020
rs10830962	rs7948640	C/T	0.89	GTEx	Cells transformed fibroblasts	SNORA32	272	C	-0.23	0.07	0.0006
rs10830962	rs7948632	C/T	0.89	GTEx	Cells transformed fibroblasts	SNORA32	272	C	-0.23	0.07	0.0006
rs10830962	rs11020123	C/T	0.87	GTEx	Cells transformed fibroblasts	SNORA32	272	C	-0.22	0.07	0.0009
rs10830962	rs7933855	G/A	0.87	GTEx	Cells transformed fibroblasts	SNORA32	272	G	-0.22	0.07	0.0008
rs10830962	rs1985254	T/C	0.86	GTEx	Cells transformed fibroblasts	SNORA32	272	T	-0.24	0.07	0.0003
rs10830962	rs2121649	C/T	0.86	GTEx	Cells transformed fibroblasts	SNORA32	272	C	-0.24	0.07	0.0005
rs10830962	rs10466351	C/T	0.86	GTEx	Cells transformed fibroblasts	SNORA32	272	C	-0.17	0.06	0.0058
rs10830962	rs11020116	A/C	0.86	GTEx	Cells transformed fibroblasts	SNORA32	272	A	-0.24	0.07	0.0003
rs10830962	rs2166706	T/C	0.84	GTEx	Cells transformed fibroblasts	SNORA32	272	T	-0.21	0.06	0.0010
rs10830962	rs724030	A/G	0.84	GTEx	Cells transformed fibroblasts	SNORA32	272	A	-0.22	0.06	0.0006
rs10830962	rs2166704	T/A	0.84	GTEx	Cells transformed fibroblasts	SNORA32	272	T	-0.21	0.06	0.0013
rs10830962	rs10765575	A/C	0.84	GTEx	Cells transformed fibroblasts	SNORA32	272	A	-0.20	0.06	0.0020
rs10830962	rs2895443	G/A	0.84	GTEx	Cells transformed fibroblasts	SNORA32	272	G	-0.23	0.06	0.0004
rs10830962	rs10830962	C/G	1.00	GTEx	Colon sigmoid	C11orf54	124	C	-0.32	0.11	0.0047

rs10830962	rs4331050	G/T	1.00	GTEx	Colon sigmoid	C11orf54	124	G	-0.32	0.11	0.0057
rs10830962	rs7941837	A/T	0.96	GTEx	Colon sigmoid	C11orf54	124	A	-0.30	0.11	0.0072
rs10830962	rs7945617	T/C	0.96	GTEx	Colon sigmoid	C11orf54	124	T	-0.30	0.11	0.0080
rs10830962	rs10830961	A/G	0.96	GTEx	Colon sigmoid	C11orf54	124	A	-0.31	0.11	0.0071
rs10830962	rs10830962	C/G	1.00	GTEx	Colon sigmoid	SCARNA9	124	C	0.36	0.13	0.0070
rs10830962	rs4331050	G/T	1.00	GTEx	Colon sigmoid	SCARNA9	124	G	0.36	0.13	0.0062
rs10830962	rs7941837	A/T	0.96	GTEx	Colon sigmoid	SCARNA9	124	A	0.36	0.13	0.0064
rs10830962	rs7945617	T/C	0.96	GTEx	Colon sigmoid	SCARNA9	124	T	0.35	0.13	0.0074
rs10830962	rs10830961	A/G	0.96	GTEx	Colon sigmoid	SCARNA9	124	A	0.36	0.13	0.0066
rs10830962	rs10830962	C/G	1.00	GTEx	Esophagus mucosa	SLC36A4	241	C	0.19	0.05	0.0002
rs10830962	rs4331050	G/T	1.00	GTEx	Esophagus mucosa	SLC36A4	241	G	0.19	0.05	0.0003
rs10830962	rs7941837	A/T	0.96	GTEx	Esophagus mucosa	SLC36A4	241	A	0.19	0.05	0.0001
rs10830962	rs7945617	T/C	0.96	GTEx	Esophagus mucosa	SLC36A4	241	T	0.19	0.05	0.0001
rs10830962	rs10830961	A/G	0.96	GTEx	Esophagus mucosa	SLC36A4	241	A	0.17	0.05	0.0005
rs10830962	rs7948640	C/T	0.89	GTEx	Esophagus mucosa	SLC36A4	241	C	0.19	0.05	0.0003
rs10830962	rs7948632	C/T	0.89	GTEx	Esophagus mucosa	SLC36A4	241	C	0.19	0.05	0.0003
rs10830962	rs11020123	C/T	0.87	GTEx	Esophagus mucosa	SLC36A4	241	C	0.19	0.05	0.0004
rs10830962	rs7933855	G/A	0.87	GTEx	Esophagus mucosa	SLC36A4	241	G	0.19	0.05	0.0003
rs10830962	rs1985254	T/C	0.86	GTEx	Esophagus mucosa	SLC36A4	241	T	0.19	0.05	0.0004
rs10830962	rs2121649	C/T	0.86	GTEx	Esophagus mucosa	SLC36A4	241	C	0.19	0.05	0.0003
rs10830962	rs10466351	C/T	0.86	GTEx	Esophagus mucosa	SLC36A4	241	C	0.20	0.05	0.0000
rs10830962	rs11020116	A/C	0.86	GTEx	Esophagus mucosa	SLC36A4	241	A	0.19	0.05	0.0002
rs10830962	rs2166706	T/C	0.84	GTEx	Esophagus mucosa	SLC36A4	241	T	0.17	0.05	0.0009
rs10830962	rs724030	A/G	0.84	GTEx	Esophagus mucosa	SLC36A4	241	A	0.17	0.05	0.0011
rs10830962	rs2166704	T/A	0.84	GTEx	Esophagus mucosa	SLC36A4	241	T	0.17	0.05	0.0011
rs10830962	rs10765575	A/C	0.84	GTEx	Esophagus mucosa	SLC36A4	241	A	0.16	0.05	0.0016
rs10830962	rs2895443	G/A	0.84	GTEx	Esophagus mucosa	SLC36A4	241	G	0.16	0.05	0.0024

rs10830962	rs7948640	C/T	0.89	GTEx	Liver	SLC36A4	97	C	0.22	0.08	0.0080
rs10830962	rs7948632	C/T	0.89	GTEx	Liver	SLC36A4	97	C	0.22	0.08	0.0079
rs10830962	rs11020123	C/T	0.87	GTEx	Liver	SLC36A4	97	C	0.23	0.08	0.0063
rs10830962	rs7933855	G/A	0.87	GTEx	Liver	SLC36A4	97	G	0.23	0.08	0.0063
rs10830962	rs2121649	C/T	0.86	GTEx	Liver	SLC36A4	97	C	0.22	0.08	0.0088
rs10830962	rs11020116	A/C	0.86	GTEx	Liver	SLC36A4	97	A	0.23	0.08	0.0066
rs10830962	rs10830962	C/G	1.00	GTEx	Nerve tibial	MED17	256	C	0.11	0.04	0.0090
rs10830962	rs7941837	A/T	0.96	GTEx	Nerve tibial	MED17	256	A	0.12	0.04	0.0044
rs10830962	rs7945617	T/C	0.96	GTEx	Nerve tibial	MED17	256	T	0.12	0.04	0.0042
rs10830962	rs10466351	C/T	0.86	GTEx	Nerve tibial	MED17	256	C	0.11	0.04	0.0035
rs10830962	rs4331050	G/T	1.00	GTEx	Ovary	SNORA18	85	G	0.24	0.09	0.0087
rs10830962	rs10830961	A/G	0.96	GTEx	Ovary	SNORA25	85	A	0.20	0.07	0.0091
rs10830962	rs10830962	C/G	1.00	GTEx	Pancreas	SNORA8	149	C	0.25	0.09	0.0078
rs10830962	rs4331050	G/T	1.00	GTEx	Pancreas	SNORA8	149	G	0.25	0.09	0.0078
rs10830962	rs7941837	A/T	0.96	GTEx	Pancreas	SNORA8	149	A	0.24	0.09	0.0082
rs10830962	rs7945617	T/C	0.96	GTEx	Pancreas	SNORA8	149	T	0.24	0.09	0.0079
rs10830962	rs10466351	C/T	0.86	GTEx	Pancreas	SNORA8	149	C	0.25	0.08	0.0036
rs10830962	rs7941837	A/T	0.96	GTEx	Pituitary	FAT3	87	A	-0.27	0.10	0.0073
rs10830962	rs7945617	T/C	0.96	GTEx	Pituitary	FAT3	87	T	-0.27	0.10	0.0074
rs10830962	rs2121649	C/T	0.86	GTEx	Pituitary	FAT3	87	C	-0.29	0.11	0.0094
rs10830962	rs10466351	C/T	0.86	GTEx	Pituitary	FAT3	87	C	-0.26	0.09	0.0066
rs10830962	rs2166706	T/C	0.84	GTEx	Pituitary	FAT3	87	T	-0.31	0.10	0.0034
rs10830962	rs724030	A/G	0.84	GTEx	Pituitary	FAT3	87	A	-0.31	0.10	0.0034
rs10830962	rs2166704	T/A	0.84	GTEx	Pituitary	FAT3	87	T	-0.30	0.10	0.0040
rs10830962	rs10765575	A/C	0.84	GTEx	Pituitary	FAT3	87	A	-0.30	0.10	0.0044
rs10830962	rs10830962	C/G	1.00	GTEx	Skin sun exposed lower leg	SNORA8	302	C	-0.12	0.04	0.0064
rs10830962	rs4331050	G/T	1.00	GTEx	Skin sun exposed lower leg	SNORA8	302	G	-0.12	0.04	0.0063

rs10830962	rs7941837	A/T	0.96	GTEx	Skin sun exposed lower leg	SNORA8	302	A	-0.12	0.04	0.0046
rs10830962	rs7945617	T/C	0.96	GTEx	Skin sun exposed lower leg	SNORA8	302	T	-0.13	0.04	0.0040
rs10830962	rs10830961	A/G	0.96	GTEx	Skin sun exposed lower leg	SNORA8	302	A	-0.12	0.04	0.0060
rs10830962	rs10466351	C/T	0.86	GTEx	Skin sun exposed lower leg	SNORA8	302	C	-0.13	0.04	0.0020
rs10830962	rs11020123	C/T	0.87	GTEx	Testis	C11orf54	157	C	-0.18	0.07	0.0086
rs10830962	rs7933855	G/A	0.87	GTEx	Testis	C11orf54	157	G	-0.18	0.07	0.0097
rs10830962	rs1985254	T/C	0.86	GTEx	Testis	C11orf54	157	T	-0.19	0.07	0.0083
rs10830962	rs2121649	C/T	0.86	GTEx	Testis	C11orf54	157	C	-0.19	0.07	0.0084
rs10830962	rs11020116	A/C	0.86	GTEx	Testis	C11orf54	157	A	-0.18	0.07	0.0091
rs10830962	rs2166706	T/C	0.84	GTEx	Testis	C11orf54	157	T	-0.20	0.07	0.0047
rs10830962	rs724030	A/G	0.84	GTEx	Testis	C11orf54	157	A	-0.20	0.07	0.0049
rs10830962	rs2166704	T/A	0.84	GTEx	Testis	C11orf54	157	T	-0.19	0.07	0.0063
rs10830962	rs10765575	A/C	0.84	GTEx	Testis	C11orf54	157	A	-0.18	0.07	0.0071
rs10830962	rs2895443	G/A	0.84	GTEx	Testis	C11orf54	157	G	-0.18	0.07	0.0092
rs10830962	rs4331050	G/T	1.00	GTEx	Thyroid	VSTM5	278	G	-0.16	0.06	0.0075
rs10830962	rs10830961	A/G	0.96	GTEx	Thyroid	VSTM5	278	A	-0.17	0.06	0.0068
rs10830962	rs11020123	C/T	0.87	GTEx	Whole blood	C11orf54	338	C	0.07	0.03	0.0094
rs10830962	rs7933855	G/A	0.87	GTEx	Whole blood	C11orf54	338	G	0.07	0.03	0.0065
rs10830962	rs1985254	T/C	0.86	GTEx	Whole blood	C11orf54	338	T	0.07	0.03	0.0052
rs10830962	rs2121649	C/T	0.86	GTEx	Whole blood	C11orf54	338	C	0.07	0.03	0.0053
rs10830962	rs11020116	A/C	0.86	GTEx	Whole blood	C11orf54	338	A	0.07	0.03	0.0091
rs10830962	rs11020123	C/T	0.87	GTEx	Whole blood	SNORA40	338	C	-0.19	0.07	0.0070
rs10830962	rs7933855	G/A	0.87	GTEx	Whole blood	SNORA40	338	G	-0.20	0.07	0.0048
rs10830962	rs1985254	T/C	0.86	GTEx	Whole blood	SNORA40	338	T	-0.20	0.07	0.0060
rs10830962	rs2121649	C/T	0.86	GTEx	Whole blood	SNORA40	338	C	-0.19	0.07	0.0083
rs10830962	rs11020116	A/C	0.86	GTEx	Whole blood	SNORA40	338	A	-0.19	0.07	0.0067
rs10830962	rs2166706	T/C	0.84	GTEx	Whole blood	SNORA40	338	T	-0.21	0.07	0.0024

rs10830962	rs724030	A/G	0.84	GTEx	Whole blood	SNORA40	338	A	-0.21	0.07	0.0024
rs10830962	rs2166704	T/A	0.84	GTEx	Whole blood	SNORA40	338	T	-0.21	0.07	0.0022
rs10830962	rs10765575	A/C	0.84	GTEx	Whole blood	SNORA40	338	A	-0.21	0.07	0.0021
rs10830962	rs2895443	G/A	0.84	GTEx	Whole blood	SNORA40	338	G	-0.21	0.07	0.0028

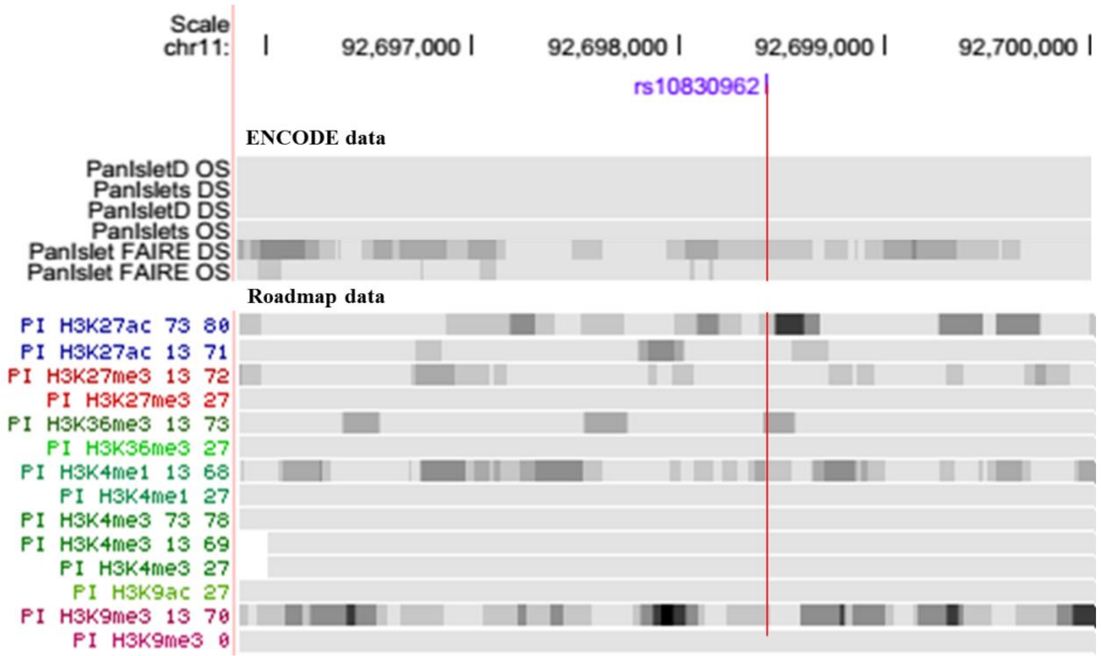

Supplementary Figure S1. Chromatin features of rs10830962.

Functional annotation in proximity to rs10830962 location in pancreatic islets (PI) from ENCODE and Roadmap data. DNaseI hypersensitivity (DS) tracks, Formaldehyde-Assisted Isolation of Regulatory Elements (FAIRE) tracks and ChIP-seq tracks (H3K27ac, H3K27me3, H3K36me3, H3K4me1, H3K4me3 and H3K9me3) for histone marks are present. The red vertical lines guide the position of rs10830962. The gray stripes represent the density signal.
